# Towards the First Principles in Biology and Cancer: New Vistas in Computational Systems Biology of Cancer

**DOI:** 10.3390/life12010021

**Published:** 2021-12-23

**Authors:** Aleš Prokop

**Affiliations:** Department of Chemical and Biomolecular Engineering, Vanderbilt University, Nashville, TN 37235-1826, USA; ales.prokop@vanderbilt.edu

**Keywords:** biology fundamentals, first principles, cancer, systems biology, non-equilibrium thermodynamics, dissipative structures, emergent properties, endogenous network hypothesis, exosomes, cancer initiation paradigms

## Abstract

These days many leading scientists argue for a new paradigm for cancer research and propose a complex systems-view of cancer supported by empirical evidence. As an example, Thea Newman (2021) has applied “the lessons learned from physical systems to a critique of reductionism in medical research, with an emphasis on cancer”. It is the understanding of this author that the mesoscale constructs that combine the bottom-up as well as top-down approaches, are very close to the concept of emergence. The mesoscale constructs can be said to be those effective components through which the system allows itself to be understood. A short list of basic concepts related to life/biology fundamentals are first introduced to demonstrate a lack of emphasis on these matters in literature. It is imperative that physical and chemical approaches are introduced and incorporated in biology to make it more conceptually sound, quantitative, and based on the first principles. Non-equilibrium thermodynamics is the only tool currently available for making progress in this direction. A brief outline of systems biology, the discovery of emergent properties, and metabolic modeling are introduced in the second part. Then, different cancer initiation concepts are reviewed, followed by application of non-equilibrium thermodynamics in the metabolic and genomic analysis of initiation and development of cancer, stressing the endogenous network hypothesis (ENH). Finally, extension of the ENH is suggested to include a cancer niche (exogenous network hypothesis). It is expected that this will lead to a unifying systems–biology approach for a future combination of the analytical and synthetic arms of two major hypotheses of cancer models (SMT and TOFT).

## 1. Introduction

Let me first describe the status of the quantitative description of biology (and cancer). Next, I will review tools from systems biology (SB). I will then discuss in more depth some nonequilibrium thermodynamic models available in literature and describe some possible ways to seek further information. For Newman (2021) see [1].

### 1.1. The Fundamental Laws of Biology/Life

Here, definitions of life and biology (and cancer) are reviewed to present the most appropriate tools for the quantitative handling of complex diseases such as cancer.

Metabolism, adaptive responses to environment, reproduction, and evolution have been recognized as fundamental characteristics of living systems [2]. Anderson [3] summarizes four principles of evolution, energy, information exchange, and systems as the basis of dealing with living systems and their components. Biology is the science of living systems and their components. The hierarchy of these systems is atomistic, subcellular, cellular, population, organ, and biome [4,5]. The principles of physics and chemistry apply at each level of the hierarchy of the living systems.

Movement towards equilibrium resulting in increasing entropy (disorder), loss of free energy, and uniformity are general characteristics of biological systems. Yet, the living systems show increasing organization. This contradiction is resolved in the form of Bauer’s principle [6] that states “The living, and only the living, systems are never in equilibrium, and, on the debit of their free energy, they continuously invest work against the realization of the equilibrium which should occur within the given outer conditions on the bases of the physical and chemical laws” [6] (p. 19).

I note that the knowledge of biological description in quantitative terms is very fragmentary and limited, except the laws of chemistry and physics. The conservative laws involve balancing mass, flux, energy, and charge. It appears that thermodynamics (non-equilibrium thermodynamics) is the only quantitative law capable of describing the biological systems.

### 1.2. Non-Equilibrium Thermodynamics in Biology (NET)

Non-equilibrium thermodynamics deals with physical systems that are not in thermodynamic equilibrium but can be described in terms of non-equilibrium state variables representing an extrapolation of the variables used to specify the system in thermodynamic equilibrium [7]. It appears that such employment results in what is called emergence in biology (see later) [8,9].

Non-equilibrium thermodynamics (also called far-from equilibrium thermodynamics) is a special case of the second law of thermodynamics. Metabolism was mentioned by Schrödinger as an example of non-equilibrium at work [10].

NET has been applied in explaining how biological organisms can develop from a disorder. Prigogine investigated methods for the thermodynamic treatment of such systems [7]. He called these systems dissipative systems because they are formed and maintained by the dissipative processes that exchange energy between the system and its environment, and because they disappear if that exchange ceases. As per the second law of thermodynamics, it maintains that any change in a closed system (i.e., a system that does not exchange any energy and mass with its environment) always results in increasing entropy. The entropy of an open system can be reduced almost to zero (in biology this is defined as death), but it is always accompanied by a greater increase in the entropy of the environment outside the open system. As a result, the entropy of the universe always increases. Biological systems do not defy this principle as the result of, and not at the expense of, increasing entropy. At the same time, dissipation structures are formed such as evolution, growth and development, differentiation, etc. (for more states, see later). As entropy increases, the information within a biological system becomes more complex or variable. This informational complexity is shaped or organized by historical, developmental, and environmental (natural selection) constraints. The complexity of biology and cancer is thus, in mathematical terms, a description of the reality of the world that often resists a description.

Another useful tool may emerge from analysis of dissipative systems. As biology does not possess physical definition, life cannot be explained on an exclusively physical basis (at the moment!). Therefore, biology is an autonomous science because it features principles that cannot be derived from the physical principles. NET attempts to describe biological processes in their time-courses in a continuous manner; thus, NET systems are spatially and temporally non-uniform. However, “their non-uniformity still has a sufficient degree of continuity (smoothness) to support the existence of suitable time and space derivatives of non-equilibrium state variables” [8,9]. Bringing time into the modeling of complex biological systems is one of the most important aspects of NET, allowing elegant solutions in the rather stiff environment of differential equations. The dimension reduction of complex biological systems leading to completely integrable partial differential equations and linearized mapping near the global solution is one of the most advantageous properties of the NET approach.

However, a new approach to the first principles was suggested by Auliffe and Nottale in 2008 [11,12]. It is based on the theory of scale relativity previously developed for physical systems by Nottale [13] to provide the required conceptual and mathematical framework to develop a theory of life based on the same first principles as physics and chemistry. The scale reference is re-interpreted here as a fractal property in terms of space–time as a new geometric tool. New principles of biology are derived from quantum-type scale laws and defined as the principles of relativity: covariance, equivalence, and geodesics. Some early derivations of this theory are based on the first law of thermodynamics.

NET is agreed upon as a principle that may “control” life. It is a commonplace belief that if the 20th century was the century of physics, the 21st century will be the century of biology, more specifically, of mathematical biology. Thermodynamics may help us to understand such complex phenomena as cancer (most of the above attributes of life can be treated, such as proliferation, cell cycle, life evolution, information transfer, angiogenesis, etc.). It provides a quantitative tool for other complex diseases and will hopefully, with a growing mechanistic knowledge (and artificial intelligence), help to crack such complex phenomena and provide new insights and improve their therapies.

In summary, most complex biological phenomena require very sophisticated tools to describe their temporal and spatial behavior. The quantification of biology (and cancer; see section below) is in very nascent form; most rules and principles are only qualitative. NET is probably the only tool available because of several advantages it provides to deal with the sophisticated system. The critique of such a continuous approach in modeling is that it cannot describe discrete data essential in cancer progression (particularly those at a fast transition between cell states—saddle points, attractors—see later). It is basically a compartmental analysis via the system of differential equations. The advantage of such a systems biology approach is that a ‘thought experiment‘ can suggest the ‘next experiment‘. 

This short list of basic concepts related to life demonstrates a lack of emphasis on these matters in literature. It is imperative that physical and chemical approaches are introduced to make biology more conceptually sound, quantitative, and based on the first principles. Non-equilibrium thermodynamics is the only tool currently available to advance the detailed description of biological phenomena in general and specifically of cancer.

## 2. Brief on Systems Biology (SB) and Discovery of Emergent Properties

Wikswo, Prokop et al. [14] defined systems biology as a “bi-directional process, linking integrative, top-down, mechanism-based or deductive modeling with bottom-up hypothesis-driven or inductive modeling. Only when inductive logic (bottoms-up model) is iteratively linked to deductive reasoning (mechanism-based) does true feedback and learning accrue.” Therefore, it is often necessary to find a middle ground methodology to systematically study complex biological and biomedical processes in the context of their experimental data. Both qualitative and quantitative tools then allow for the evaluation of relationships and interactions among various system components, typically under multiscale and dynamic conditions. The result of this effort is typically an integrated phenotype at the whole-body level, which will, by definition, include lower-level hierarchies at the molecular, cellular, tissue and organ levels. In a clear departure from the mechanism-based reductionist approach, SB embraces both arms of scientific dogma, reductionist and holistic, by employing an integrated, closed-loop learning middle ground” [4]. As more SB global tools are developed and better extraction of emergent properties is instituted, Biomedical R&D will experience a paradigm shift to this more systemic approach. “By closing the loop between induction and deduction in a systematic, modeling context, the field will gain a deeper understanding of disease mechanisms (including the upper-level interactions)” [14]. It also worth citing a book on systems biology by Bizzari M [15], which provides the methodological aspects of systems biology, including step-by-step laboratory protocols and a primer on mathematical modeling in biology.

SB aims to develop and use efficient algorithms, data structures, visualization, and communication tools to integrate large quantities of biological data with the goal of computer modeling. The edited book by Prokop and Csukas [16] presented tools for handling of big data, nonlinearity, and hidden interactions in systems biology. Other basic tools presented there include: graph classifying of networks, emergent properties of networks, regulatory crosstalk analysis, network analysis, computational tools, probabilistic graphical modeling, rule-based modeling, parameter identifiability and redundancy, semantic systems biology, agent-based modeling, reconstruction of cellular signaling pathway, integrative network algorithms, and direct computer mapping. A network is any system (in our case a biological one) with sub-systems interconnected into a whole. Some other terms are explained in the text below.

The section on SB is introduced here because cancer is a disease based on a malfunctioning of the system properties and features an aberrant signaling and progression, hence a systems biology disease [17,18,19]. Indeed, progress in cancer research toward cancer therapy may develop faster if cancer is studied not only in terms of molecular biology but also in terms of systems. The central dogma of SB is that it is the dynamic interactions of molecules and cells that give rise to biological function (emergent property) via computational modeling to reconstruct complex systems from a wealth of reductionist, molecular data (e.g., gene/protein expression, signal transduction activity, metabolic activity, cell–cell interactions, etc.). Thus, both arms of exploration are needed, absolutely. Further on, emergent property will be defined and some references will be presented for gene and metabolic systems.

Many phenomena at subcellular/cellular levels that are typically described in biochemistry and cell biology textbooks can be considered as phenomena resulting from the interaction between different compartments and hierarchies (e.g., cell growth, division/proliferation, apoptosis, cell activation, homeostasis, cell death, differentiation, system robustness, redundancy, multiplicity of steady-states, hysteresis, oscillations, structural hierarchy, consciousness, self-organization, evolution, self-awareness, etc.). The latter hierarchies probably fall into the category of first principles in biology. Venkatasubramanian [20] stated that “many grand challenges that we face in 21st century science are bottom-up phenomena, going from the parts to the whole”. Examples in biology include predicting a phenotype from a genotype, predicting the effects of human behavior on the global climate, and predicting consciousness and self-awareness, quantitatively and analytically. The scope of computational research should be to redefine such properties in terms of mechanisms and quantify them via the SB approach. That is, to elucidate these properties in terms of interacting species and topologies of lower levels. The systems view then dictates that “one should target network state (while seeking a drug) resulting from gene/protein networks (see below, ‘dissipative structures’) and their emergent behavior, rather than individual genes or proteins as a new strategy for drug discovery, suggesting that we explore multi-target drugs or non-additive combination therapies” [20].

Complexity is a property of systems with interacting parts. When the interactions are nonlinear, it is not possible to reduce the system’s behavior to a simple sum of those parts. Closely related to complexity is the concept of emergence [21], mentioned previously. “Emergence is generally taken to mean simply that the whole is more than the sum of its parts, or that system-level characteristics are not easily derivable from the local properties of their constituents” [22]. This implies that although higher-level phenomena are not reducible to physical laws, they may still be consistent with them. “This multiple scale causality not only recognizes multiple processes and controls acting at multiple scales but, unlike a strict reductionist approach, may also recognize the fact that relevant ‘first principles’ may reside at scales other than the smallest micro-scales. In other words, the observed phenomenon at each scale has structural and behavioral properties that do not exist at lower or higher organizational levels. Therefore, the modeling of some biological processes cannot solely follow a bottom-up approach; they must eventually include “high-level organizing principles and even downward causality” [23]. As such, complex systems are hard to analyze using traditional mathematical and analytical methods. Despite this difficulty, emergence can be studied and revealed computationally. Richards et al. [23] presented a simple two-dimensional molecular dynamics lattice model for interacting chemical species capable of generating higher-order emergent properties (EPs). The concept of emergence may serve as one of the most unifying themes across scientific disciplines, notably in biology.

EPs are understood to be integrated network properties that are non-obvious and impossible to derive intuitively from a simple inspection of the reaction network. At a higher level of organization, an interaction between modules, in mathematical terms, can produce novel behavior. Thus, e.g., the immune system has been modeled as a complex system, exhibiting emergent properties [24], as well as cell size at S phase initiation (cell cycle) as an emergent property while modeling mammalian proliferation [25]. The phenomenon of emergence, its quantitative description, is the single most important benefit of computational systems biology. Its refinement will become the subject of this century.

## 3. Brief on Metabolic Models and Metabolic Engineering

Traditionally, metabolic engineering is used for optimizing genetics and regulatory processes in organisms. In systems biology, the metabolic pathway is mathematically treated to arrive at reaction rates, resulting in flux balance analysis (FBA) [26,27]. It was applied in the genome-scale metabolic network reconstructions that were built in the past decade. These networks contain all the known metabolic reactions in an organism and the genes that encode each enzyme. FBA calculates the rates of change of metabolites through this metabolic network. The advantage of an attempt to reconstruct genome-scale metabolic systems is that it often leads to discoveries of new reactions missing from our standard approach. FBA compares in silico system simulations to experimental results [27], and as such it is an indispensable tool in cancer biology.

## 4. Multiscale Simulation

The complex mechanisms in biology occur at several spatial and temporal scales. Various methods have been developed for solving multi-scale problems in many scientific disciplines and applied to biological systems. New computational tools are available to solve various aspects of cancer biology [28,29,30,31,32].

All of the above should be considered as an introduction to the rest of the manuscript below, which is the essential part of this manuscript.

## 5. Cancer Concepts

To introduce this section, the main goal of this article is to draw attention to the biology of cancer and its connection to existing knowledge of the laws of physics and chemistry and to what the researchers have missed by not realizing the nature of live biological systems to fight equilibrium (this is what sets a biological system apart from its physical and chemical components, whose tendency is always to move toward equilibrium).

### 5.1. Cancer Hallmarks—SMT Paradigm

**Figure 1:**
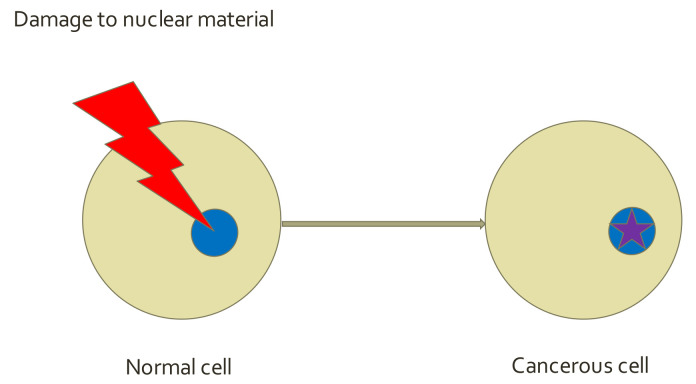
STM paradigm. Random mutation occurs to the original cell (blue object), not to the environment.

I now introduce the qualitative concept of “The Hallmarks of Cancer” as published by Douglas Hanahan and Robert Weinberg in 2000 and extended in 2011 [33,34]. These authors believe that the complexity of cancer can be reduced to a small number of underlying principles. The first paper argues that “all cancers share six to nine common traits (‘hallmarks’) that govern the transformation of normal cells to cancer (malignant or tumor) cells.” Such a generalization is allowed as all cancers might share basic elements of a disease state despite the great diversity of cancer disease states. Furthermore, this concept assumes that cancer cells are generated by random mutation focused on the original healthy cell, not the tissue surroundings (this is known as the somatic mutation theory; see SMT paradigm, Figure 1). However, this approach is slowly being replaced by the tissue organization field theory of cancer (TOFT) [35] and by some newer paradigms (see below), stressing the environmental (external) effects.

### 5.2. Tissue Organization Field Theory of Cancer (TOFT) and Related Concepts

**Figure 2:**
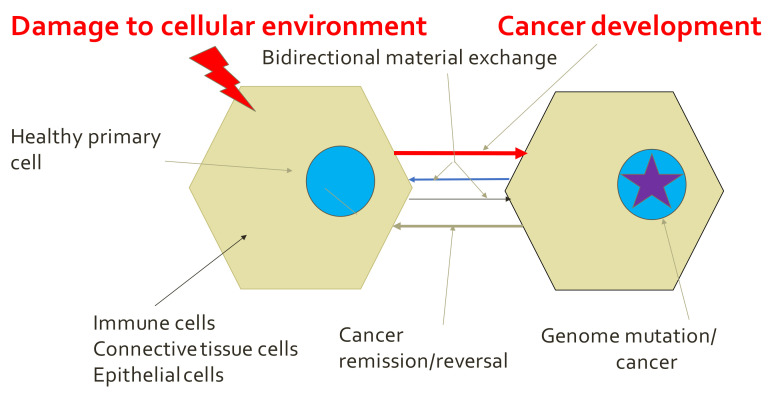
TOFT paradigm. Cancer occurs from the disorder of the microenvironment (represented by hexagonal gray object).

The TOFT theory is system-oriented as compared to Weinberg–Hanahan’s theory, which is reductionist. TOFT “suggests that cancer results from a disorder of the microenvironment of the cell that represents the physicochemical support by the morphogenetic field that drives epithelial cells (Figure 2). This involves complex and reciprocal (bidirectional) biophysical, and biochemical communication between mesenchymal (stromal or connective tissue) and parenchymal (epithelial) cells” [36]. The authors claimed that cancer is caused by a sustained failure of communication between the interacting cell lineages living in the complex cellular society of an organ, and thus of regulatory networks, within the field of the affected tissues [37]. This new TOFT paradigm opens the possibility of targeting specific tissue interactions between tumor cells and their non-neoplastic neighbors that sustain tumor cells, as well as targeting the tumor cells themselves. However, many multiple specific aspects of biology, physiology, and biochemistry as well as different wound healing processes, signaling paths, etc., have not yet been included within the TOFT paradigm. Nevertheless, both paradigms will be used in this review, as each offers something valuable. We should be aware of the existence of these two concepts (SMT and TOFT) to interpret our experiments in more realistic way. The TOFT concept is largely qualitative. The concept of reciprocal communication between the tumor and its environment is briefly discussed below. The importance of the microenvironment in tumor development and progression is widely recognized. The tumor microenvironment is composed of both tumor cells and stromal cells. Persistent crosstalk between tumor cells and stromal cells is required for tumor growth and metastasis, intercellular communication between the tumor microenvironment and cancer cells [38]. Emerging evidence suggests that exosomes, the small particles derived from cells with important biological functions, play a key role in the tumor–stromal interaction. Exosomes may release many important tumor cell components, such as proteins, nucleic acids, and lipids that can be transmitted to recipient cells. Cancer cells actively communicate with the tumor environment through exosomes, which constitutes a bidirectional interaction network to synergistically interact between the tumor cells and stroma/microenvironment [39].

### 5.3. Epistemological Origin of the Cancer Paradigm

**Figure 3:**
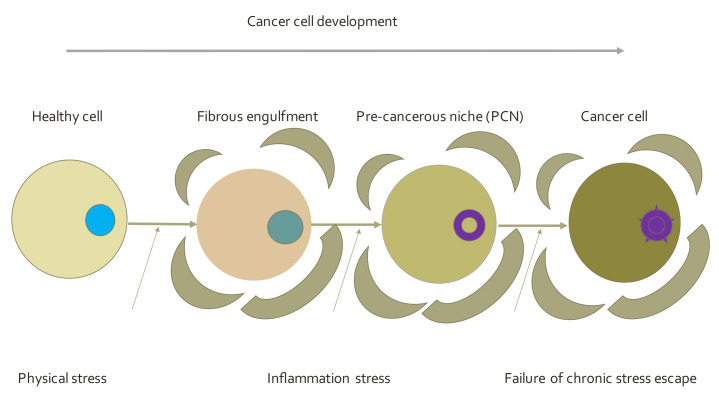
Epistemological origin of the cancer paradigm consists of pathogenic stress, chronic inflammation, pre-cancerous niche, chronic stress escape strategy, and cancer cell status.

Still another notable paradigm was recently proposed advocating a multistep detailed sequence of events creating a precancerous niche (PCN) before arriving at a cell transition as formulated by Brűcher [40,41] (Figure 3). This new cancer hypothesis also provides the necessary biochemical and physiological signaling pathways [42] and consists of (1) a pathogenic stimulus (biological or chemical) followed by (2) chronic inflammation, from which develops (3) fibrosis with associated changes in the cellular microenvironment. From these changes a (4) pre-cancerous niche develops, which triggers the deployment of (5) a chronic stress escape strategy, and, when this fails to resolve, (6) the transition of a normal cell to a cancer cell occurs. The authors additionally provided strong evidence for why the emphasis on genetics and epigenetics [42] has so far failed to make a clinically significant difference in cancer treatment, and suggested why a new anti-cancer strategy is needed [43]. According to the authors, the majority of cancers do not originate by mutations, and the findings in cancer genetics so far reported are either late events or are epiphenomena that occur after the appearance of the pre-cancerous niche [44,45]. This model indicates a “need to establish preventive measures long before a cancer becomes clinically apparent.” Future research should focus on the intermediate steps in the proposed sequence of events, which will enhance our understanding of the nature of carcinogenesis, which should focus on suppressing the multistep sequences including the PNC with early intervention. As demonstrated by Gao et al. this step may involve the effect of exosomes (derived from different donor cells, e.g., T cells) in premetastatic niche formation in gastric cancer [46]. The presence of PNC should detect and quantify any subclinical inflammatory change to treat all levels of pathogenic chronic inflammation and prevent fibrotic changes, and so avoid the transition from a normal cell to a cancer cell. This concept is qualitative only. However, many of the sequences mentioned above are amenable to a quantitative treatment.

### 5.4. Endogenous Network Hypothesis (ENH)—Far-from Equilibrium Dynamical Systems Theory of Cancer

The ENH is also denoted as far-from thermodynamic equilibrium (“off-equilibrium”). Ao et al. [47,48] suggested that cancer corresponds to an emergent robust state from interactions among endogenous molecules. I note that this theory is about 10 years old, and still, I do not see the acceptance of this theory by cancer scientists. Later, I will indicate how to get over this hurdle.

**Figure 4:**
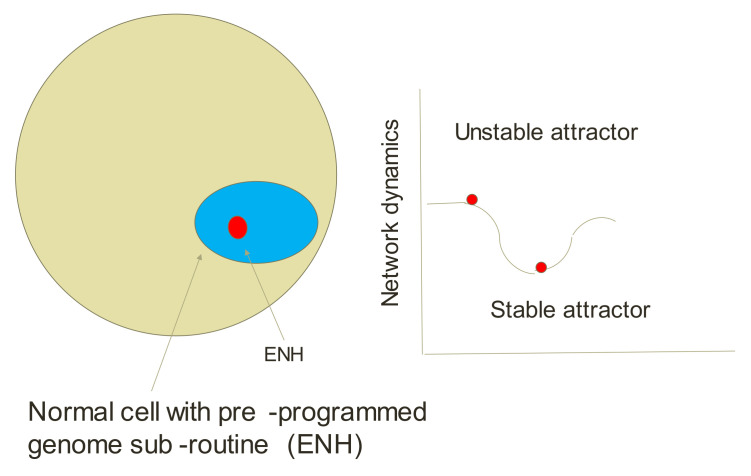
Endogenous network hypothesis (ENH). Environment changes the network dynamics (two positive feedbacks) from an unstable to stable (cancerous) attractor site.

Carcinogenesis in the ENH may be understood as a transition from a normal state to cancer state(s) in a potential landscape (Figure 4). The potential landscape is a quantitative realization of pioneering ideas: Wright’s adaptive landscape [49] and Waddington’s developmental landscape [50]. Perturbations such as environmental changes may lead to a potential reshaping and eventually to a cancerous state.

In this theory, the endogenous molecular network consists of several endogenous factors and their interaction network. The activity/concentration of each endogenous factor is modulated by other endogenous factors via signal transduction and transcriptional relations. The endogenous factors and their activation/upregulation and inhibition/downregulation relations can be drawn from documented signaling transduction pathways and gene transcription networks available in literature. This concept employs a set of standard chemical rate equations that includes production rate, degradation rate, and feedback loops.

It should be noted that the present endogenous network of cancer is a tremendously simplified framework. The endogenous molecular network includes both genetic information and biochemical interactions among endogenous factors. A detailed molecular endogenous network should contain in future a vast number of endogenous factors, such as microRNA, metabolites, and other proteins. At present, “the modeling of all interactions remains beyond our capabilities” [51]. The ENH concept was further developed in a subsequent paper by Ping Ao’s group (e.g., [52]). Huang et al. [53] expanded on the term of attractor state previously proposed by Kaufman as “a set of numerical values toward which a system tends to evolve, for a wide variety of starting conditions of the system.” The nonlinear dynamical interactions among the endogenous agents can generate many local attractors with obvious or non-obvious biological functions. Cancer is one of the attractor states. In light of the present hypothesis of Ao, the genesis and regression of cancer are viewed as transitions between normal tissue and cancer attractors passing through saddle points or other attractors. The term regression is emphasized here as a possible state to establish a healthy state. As such it is built into the ENH concept (see below).

The dynamics of the endogenous network is autonomous. The network can generate many locally stable states with obvious or non-obvious biological functions. Metastable states that have a sufficiently large, attractive basin are insensitive to structural perturbations and are identified as robust states. Cancer and normal functions correspond to different intrinsic robust states. In addition, stochastic transitions are bidirectional, since there are spontaneous transitions to a cancer state; the transition from the cancer state to a normal healthy state is also possible, which naturally explains the spontaneous regression of cancer. Other groups have also suggested a similar concept to the ENH (e.g., [54]—the landscape and flux theory).

Yuan et al. [55] and Wang et al. [56] expanded on the original ENH. Several core networks have been constructed for several examples of disease. It is of interest that the network geometry overlaps strongly among different disease models. The endogenous network theory generates distinct predictions qualitatively different from those of the somatic mutation theory, such as the rise of cancer-like cells (or neoplasms) without a genetic defect and the existence of spontaneous remission/regression of cancer patients without a treatment. Moreover, the framework for decomposing the general stochastic dynamics to obtain a potential landscape enables computing multiple normal cell types. All the above result from the theory of non-equilibrium thermodynamics. A natural question would be: Can the endogenous molecular–cellular network model generate all normal cell types that emerge in the developmental processes? Cellular evolution and stem cell differentiation are good examples (e.g., [57]). It can be inferred that, based on evidence from evolution, a comprehensive and evolutionarily conserved endogenous network may contain several hundreds of nodes (at present only about 50 gene nodes have been employed at simulations; note: node is defined as a point at which pathways intersect or branch or as a hub or connector). More biological experiments are needed to define such nodes. The ultimate goal is the construction of a unique genome-scale endogenous molecular–cellular network model that can generate many robust states, including known cell types, functional states, as well as diseases; the ENH is thus the unification theory, quantitative in design. In summary: this concept suggests “the existence of a hierarchical structure within, molecular biology systems. The cancer status (for a healthy individual) is built into the molecular (gene) regulatory networks (GRN): such networks can be generated from a good number of key genes (signaling networks) involved in cancer. There is even built in a precancerous state of cells and path to differentiation and finally to cancer (such as normally dormant, tissue-restricted or cryptic enhancers or promoters that serve to drive oncogenic gene expression)” (for signaling pathways, see, e.g., [58,59,60,61,62]). In addition, the environmental cues (a tumor microenvironment consisting of different cells plus immune cells) are there if the GRN is properly formulated. Heng [63] summarized the above by stating that “Cancer is a pre-programmed, well-organized and survival response to a threatening (corrected!) cellular microenvironment. All cells have this pre-loaded ancient ‘cancer subroutine’”. This ontogenically developed, pre-programmed cancer potential is not active under normal circumstances; however, it can be activated as part of the neoplasms’ survival response. In other words, as stated by Plutynski and Bertolaso [64], cancer results from “a robust state of the endogenous cellular network”. That is, “our vulnerability to cancer is an intrinsic property of the cells—one inherited from the developmental and evolutionary history of the organism”.

The ENH is the most intriguing hypothesis of all mentioned above. An explosive increase in our experimental data on cancer networks (number of nodes–genes) will provide more evidence that the non-equilibrium thermodynamics approach may yield constructs that will be much closer to the reality. This concept is similar to that of dissipative system and emergent property.

Following their initial discovery of the ENH, Ao and his team in later years presented a rigorous theoretical work and launched extensive experimental studies on individual cancers [65,66]. In 2010 [65] Ao provided enumeration of this theory using stochastic dynamic equations. The final diagram consisted of 37 members. This hypothesis was applied to study prostate, liver, and gastric cancers, and surprisingly, all cases fitted well with the same core composition [65]. The network resulted in a landscape consisting of many locally (robust) stable normal states and several unhealthy states with no obvious function [52].

Further considerations were also put forward based on the NET theory. Hanselmann and Welter [67] stated that cancer cells are thermodynamically far away from equilibrium and possess three characteristics: the energy loss (e.g., through mitochondrial dysfunction), information disturbances (i.e., aneuploidy), and changes in the matter (e.g., through micro-environmental stress), leading to irreversibly imbalanced but thermodynamically more stable cells. Mutation as the only cause of cancer has been rejected based on this complex interplay of energy, information, and the matter, presented only in a qualitative way. Another addition worth mentioning is that the Ping Ao’s endogenous core protein/gene is constant over the cancer development [52], Li and Wang’s [68] endogenous gene set changes during this stage, which is more realistic. In addition, their method uses a set of mRNAs as well as relevant genes.

The computation within complex networks of agents is likely to become the leading research area during the 21st century [69].

## 6. Future in Cancer Genomics by the ENH Method

There are several extensions of the NET theory I can put forward: (i) the separate effect of the exogenous gene network (EGN) covering a cancer niche (the microenvironment, including the immune system, covering both arms of immunity); this step remains to be explored in the near future; (ii) effect of the competing endogenous RNA network (ceRNA, microRNA; combined NET plus CER), and (iii) application of the ENH to provide a theory of disease initiation and development, a disease gene network (DGN), for those diseases of genetic background. Other extensions may include competing endogenous RNAs, mRNAs, long noncoding RNAs, circular RNA and microRNA [70,71,72]. Their function could be further analyzed by the ENH method. According to Heng [64] the ENH could also be extended to the tumor microenvironment, that is, with the existence of a pre-disposed silent cancerous state but this time derived from the microenvironment.

## 7. Conclusions—An Ultimate Goal—Comprehensive E-Pharma Cancer Model

While reviewing the above cancer paradigms, I propose entertaining both reductionist and organismic approaches such as TOFT and SMT as they do not contradict each other but come into confluence and may complement each other in a single unified theory of carcinogenesis [73]. A fusion of both reductionist and systems biology concepts was suggested by Huang [74,75] based on the dynamical systems theory featuring the far-from equilibrium (thermodynamic) network biology. Particularly important is an intercellular horizontal interaction between the stroma and cancer milieu. Likewise, Yuan et al. [76] and Li and Wang [70] reconstructed a cancer regulatory network from literature and demonstrated that this far-from equilibrium model provides an embedded normal cell state, cancer state, and apoptosis depending on the degree of perturbation, as discussed earlier. Such an approach could be extended to include both genomic and micro-environmental inputs. I believe that the thermodynamic approach extended to a large set of genomic and cellular data as discussed above will provide a unifying approach for future construction of a comprehensive E-Pharma cancer model, which could be entertained for drug discovery and development at an industrial scale. NET has been employed to describe many complex facets of biology, beginning with the origin of life and ending with cell phenomena, cell differentiation, consciousness, and ecology. I can certainly add a new hallmark of cancer—a thermodynamic signature [63,77]. A breakthrough in statistical physics for the description of biological systems is urgently needed. The latest addition to the topic discussed in this review is worth mentioning [78].

Concluding, the systems biology approach appears as the only tool for quantifying the phenomena in cancer. The inclusion of the cancer hierarchy (emergent properties) is necessary to guarantee further progress (e.g., inclusion of immune cells and exosome effects). However, further progress in the practical utilization of the systems approach will be only useful when the upper echelons of hierarchy are included in the ENH applications as well.

## References

[1] Newman T., Bertolaso M., Strauss B. (2021). Cancer as a System Hard Lessons from Physics and a Way Forward. The Search for Progress and a New Theory Framework in Cancer Research.

[2] Rasmussen S., Baas N.A., Mayer B., Nilsson M. (2001). M Defense of the ansatz for dynamical hierarchies. Artif. Life.

[3] Andersen P. http://www.bozemanscience.com.

[4] Prokop A. (1982). Systems analysis and synthesis in biology and biotechnology. Int. J. General. Syst..

[5] Prokop A., Michelson S. (2009). Systems Biology in Biotech and Pharma.

[6] Bauer E. Theoretical Biology.

[7] Prigogine I. (1967). Introduction to Thermodynamics of Irreversible Processes (1955/1961/1967).

[8] Steely A.J.E., Macklen P. (2012). Fractal variability: An emergent property of complex dissipative systems. Chaos.

[9] (2004). Davies PCW Emergent biological principles and computational properties of the Universe. Complexity.

[10] Schrödinger E. (1967). What is Life?.

[11] Nottale L., Auffray C. (2008). Scale relativity theory and integrative systems biology: 2. Macroscopic quantum-type mechanics. Prog. Biophys. Mol. Biol..

[12] Auffray C., Nottale L. (2008). Scale relativity theory and integrative systems biology: 1. Founding principles and scale laws. Prog. Biophys. Mol. Biol..

[13] Nottale L. (1989). Fractals and the quantum theory of spacetime. Int. J. Mod. Phys. A.

[14] Wikswo J.P., Prokop A., Baudenbacher F.J., Cliffel D., Csukas B., Velkovsky M. (2006). The engineering challenges of BioMEMS: The integration of microfluidics, micro- and nano-devices, models, and external control for systems biology. IEE Proc. Nanobiotechnol..

[15] Bizzarri M. (2018). Editor: Systems Biology.

[16] Prokop A., Csukas B. (2013). Systems Biology. Integrative Biology and Simulation Tools.

[17] Hornberg J.J., Bruggeman F.J., Westerhoff H.V., Lankelma J. (2006). Cancer: A systems biology disease. BioSystems.

[18] Kreeger P.K., Lauffenburger D.A. (2010). Cancer systems biology: A network modeling perspective. Carcinogenesis.

[19] Korsunsky I., McGovern K., LaGatta T., Olde Loohuis L., Grosso-Applewhite T., Griffeth N., Mishra B. (2014). Systems biology of cancer: A Challenging expedition for clinical and quantitative biologists. Front. Bioeng. Biotechnol..

[20] Venkatasubramanian V. (2019). The promise of artificial intelligence in chemical engineering: Is it here, finally?. AIChE J..

[21] Buchanan M. (2006). Nexus: The Groundbreaking Science of Networks.

[22] Fromm J. (2008). Ten questions about emergence. arXiv.

[23] Richards K., Bithell M., Dove M., Hodge R. (2004). Discrete-element modelling: Methods and applications in the environmental sciences. Philos. Trans. R. Soc. A Math. Phys. Eng. Sci..

[24] Ahmed E., Hashish A.H. (2006). On modelling the immune system as a complex system. J. Theor. Biol..

[25] Barberis M., Klipp E., Vanoni M., Alberghina L. (2007). Cell size at S phase initiation: An emergent property of the G1/S network. PLoS Comput. Biol..

[26] Varma A., Palsson B.O. (1994). Stoichiometric flux balance models quantitatively predict growth and metabolic by-product secretion in wildtype Escherichia coli W3110. Appl. Environ. Microbiol..

[27] Papin J.A., Palsson B.Ø. (2004). Topological analysis of mass-balanced signaling networks: A framework to obtain network properties including crosstalk. J. Theor. Biol..

[28] Dada J.O., Mendes M. (2011). Multi-scale modelling and simulation in systems biology. Integr. Biol..

[29] Wolkenhauer O., Auffray C., Brass O., Clairambault J., Deutsch A., Drasdo D., Gervasio F., Preziosi L., Maini P., Marciniak-Czochra A. (2014). Enabling multiscale modeling in systems medicine. Genome Med..

[30] Eissing T., Kuepfer L., Becker C., Block M., Coboeken K., Gaub T., Goerlitz L., Jaeger J., Loosen R., Ludewig B. (2011). A com-putational systems biology software platform for multiscale modeling and simulation: Integrating whole-body physiology, disease biology, and molecular reaction networks. Front. Physiol..

[31] Chevalier M.W., El-Samad H. (2009). A rigorous framework for multiscale simulation of stochastic cellular networks. J. Chem. Phys..

[32] Pérez-Velázquez J., Gevertz J.L., Karolak A., Rejniak K.A., Rejniak K. (2016). Microenvironmental Niches and Sanctuaries: A Route to Acquired Resistance. Systems Biology of Tumor Microenvironment.

[33] Hanahan D., Weinberg R.A. (2000). Hallmarks of cancer. Cell.

[34] Hanahan D., Weinberg R.A. (2001). Hallmarks of cancer: The next generation. Cell.

[35] Sonnenschein C., Soto A.M., Rangarajan A., Kulkarni P. (2014). Competing views on cancer. J. Biosci..

[36] Smythies J. (2005). Intercellular signaling in cancer-the SMT and TOFT hypotheses, exosomes, telocytes and metastases: Is the messenger in the message?. J. Cancer.

[37] Macklin P., Lowengrub J. (2007). Nonlinear simulation of the effect of microenvironment on tumor growth. J. Theor. Biol..

[38] Valcz G., Buzás E., Szállási Z., Kalmár A., Krenács T., Tulassay Z., Igaz P., Molnár B. (2018). Perspective: Bidirectional exosomal transport between cancer stem cells and their fibroblast-rich microenvironment during metastasis formation. Breast Cancer.

[39] Lu M., Huang B., Hanasch S.M., Onuchica J.N., Ben-Jacoba E. (2014). Modeling putative therapeutic implications of exosome exchange between tumor and immune cells. Proc. Natl. Acad. Sci. USA.

[40] Brűcher B.L.D.M., Jamall I.S. (2014). Epistemology of the origin of cancer: A new paradigm. BMC Cancer.

[41] Bishop R.C., Hooker C. (2001). Metaphysical and epistemological issues in complex systems. Handbook of the Philosophy of Science.

[42] Brücher B.L.D.M., Jamall I.S. (2014). Cell-Cell communication in tumor microenvironment, carcinogenesis and anticancer treatment. Cell. Physiol. Biochem..

[43] Brücher B.L.D.M., Li Y., Schnabel P., Daumer M., Wallace T.J., Kube R., Zilberstein B., Steele S., Voskuil J.L., Jamall I.S. (2016). Genomics, microRNA, epigenetics, and proteomics for future diagnosis, treatment and monitoring response in upper GI cancers. Clin. Transl. Med..

[44] Brücher B.L., Lyman G., van Hillegersberg R., Pollock R.E., Lordick F., Yang H.K., Ushijima T., Yeoh K.G., Skricka T., Polkowski W. (2014). Imagine a world without cancer. BMC Cancer.

[45] Jamall I.S. (2016). Somatic mutation theory—Why it’s wrong for most cancers. Cell. Physiol. Biochem..

[46] Gao J., Li S., Xu Q., Zhang X., Huang M., Dai X., Liu L. (2021). Exosomes promote pre-metastatic niche formation in gastric cancers. Front. Oncol..

[47] Ao P., Kwon C., Qian H. (2001). On the existence of potential landscape in the evolution of complex systems. Complexity.

[48] Ao P., Galas D., Hood L., Zhu X. (2008). Cancer as robust intrinsic state of endogenous molecular-cellular network shaped by evolution. Med. Hypothesis.

[49] Wright S. The roles of mutation, inbreeding, crossbreeding, and selection in evolution. Proceedings of the Sixth International Congress of Genetics.

[50] Waddington C.H. (1957). The Strategy of the Genes.

[51] Pe’er D., Hacohen N. (2011). Principles and strategies for developing network models in cancer. Cell.

[52] Wang G., Yuan R., Zhu X., Ao P., Bizzarri M. (2018). Endogenous molecular-cellular network cancer theory: A systems biology approach. Systems Biology, Methods in Molecular Biology.

[53] Huang S., Ernberg I., Kauffman S. (2009). Cancer attractors: A systems view of tumors from a gene network dynamics and developmental perspective. Semin. Cell Dev. Biol..

[54] Wang J. (2015). Landscape and flux theory of non-equilibrium dynamical systems with application to biology. Advan. Phys..

[55] Yuan Y., Liu B., Peng X.P., Zhang M.Q., Li Y., Xie Z., Wang X. (2015). Model-guided quantitative analysis of microRNAmediated regulation on competing endogenous RNAs using a synthetic gene circuit. Proc. Natl. Acad. Sci. USA.

[56] Wang J., Xu L., Wang E., Huang S. (2010). The potential landscape of genetic circuits imposes the arrow of time in stem cell differentiation. Biophys. J..

[57] Kauffman S. (2017). Differentiation of malignant to benign cells. J. Theor. Biol..

[58] Ochsner S.A., Abraham D., Martin K., Ding W., McOwiti A., Kankanamge W., Wang Z., Andreano K., Hamilton R.A., Chen Y. (2019). The Signaling Pathways Project, an integrated omics knowledge base for mammalian cellular signaling pathways. Sci. Data.

[59] Sever R., Brugge J.S. (2015). Signal transduction in cancer. Cold Spring Harb. Perspect. Med..

[60] Staal F.J., Famili F., Garcia Perez L., Pike-Overzet K. (2016). Aberrant Wnt Signaling in Leukemia. Cancers.

[61] Tocris Biosciences. https//www.tocris.com/signaling-pathways.

[62] ThermoFisher. https://www.thermofisher.com.

[63] Heng H.H. (2016). Alternative theories to explain cancer, Chapter 3. Debating Cancer.

[64] Plutynski A., Bertolaso M. (2018). What and how do cancer systems biologists explain?. Philos. Sci..

[65] Ao P., Galas D., Hood L., Yin L., Zhu X.M. (2010). Towards predictive stochastic dynamical modeling of cancer genesis and progression. Interdisc. Sci..

[66] Ao P. (2016). Endogenous network hypothesis for cancer genesis and progression. Eur. J. Cancer.

[67] Hanselmann R.G., Welter C. (2016). Origin of cancer: An information, energy, and matter disease. Front. Cell Dev. Biol..

[68] Li C., Wang J. (2015). Quantifying the underlying landscape and paths of cancer. J. R. Soc. Interface.

[69] Hooker C.A., Hooker C. (2011). Philosophy of Complex Systems.

[70] Anastasiadou E., Jacob L.S., Slack F.J. (2018). Non-coding RNA networks in cancer. Nat. Rev. Cancer.

[71] Zhang W., Bojorquez-Gomez A., Velez D.O., Xu G., Sanchez K.S., Shen J.P., Chen K., Licon K., Melton C., Olson K.M. (2018). A global transcriptional network connecting noncoding mutations to changes in tumor gene expression. Nat. Genet..

[72] Shabalina S.A., Spiridonov N.A. (2004). The mammalian transcriptome and the function of non-coding DNA sequences. Genome Biol..

[73] Rosenfeld S. (2013). Are the somatic mutations and tissue organization field theories of carcinogenesis compatible?. Cancer Informat..

[74] Huang S. (2012). Tumor progression: Chance and necessity in Darwinian and Lamarckian somatic (mutationless) evolution. Prog. Biophys. Mol. Biol..

[75] Huang S., Li F., Zhou J.X., Qian H. (2017). Processes on the emergent landscapes of biochemical reaction network and heterogenous cell population dynamics: Differentiation in living matters. J. R. Soc. Interface.

[76] Yuan R., Zhu X., Wang G., Li S., Ao P. (2017). Cancer as robust intrinsic state shaped by evolution: A key issues review. Rep. Prog. Phys..

[77] Remacle F., Zadran S. (2013). Can Thermodynamics Help Us Better Understand Human Cancers?.

[78] Bertolaso M., Strauss B. (2021). The Search for Progress and a New Theory Framework in Cancer Research. Rethinking Cancer. A New Paradigm for Postgenomics Era. Vienna Series in Theoretical Biology.

